# A Neoteric Paradigm to Improve Food Security: The Predictors of Women’s Influence on Egocentric Networks’ Food Waste Behaviors

**DOI:** 10.3390/nu16060788

**Published:** 2024-03-10

**Authors:** Karissa Palmer, Robert Strong, Chanda Elbert

**Affiliations:** Department of Agricultural Leadership, Education and Communications, Texas A&M University, College Station, TX 77843, USA; karissa.palmer@ag.tamu.edu (K.P.); chanda.elbert@ag.tamu.edu (C.E.)

**Keywords:** Generation X, Farm Bureau, opinion leadership, advice networks, food sustainability, food systems, sustainable development goals (SDGs), no poverty, zero hunger, sustainable communities

## Abstract

COVID-19, the most recent multi-dimensional global food crisis, challenged leadership and impacted individuals’ personal networks. Two cross-sectional surveys were disseminated to women involved in their state’s women’s leadership committee to understand food waste behaviors. An egocentric network analysis was chosen as the methodology to better understand personal advice network characteristics and examine the impacts of Farm Bureau women’s leadership committee members’ advice networks on their food waste behavior. A multilevel model was conducted to identify factors related to respondents leading their network members toward positive food waste decisions. Independent variables included in the variables at the individual (e.g., each respondent’s race, generation), dyadic (e.g., length respondent has known each member of her network), and network levels (e.g., proportion of the respondent’s network that was female) were included in the model. Women were more likely to report connections with people they led to positive food waste behaviors and food security when: they had higher food waste sum scores, they were part of Generation X, the network member they led to more positive food waste behaviors was a friend, and if there were fewer women in their advice networks.

## 1. Introduction

The world has faced several unforeseen crises, forcing leaders to make timely and confident decisions [1]. The most recent global crisis, the COVID-19 pandemic, continues to impact the agricultural industry, prompting organizations to make changes to their strategies to understand these impacts [2]. The COVID-19 virus itself is not the current threat to food security, it is the loss of income and buying power due to the lockdown forced by national and local governments [3]. COVID-19 has negatively affected already-vulnerable and marginalized populations’ access to safe and nutritious foods [4].

The global population is forecasted to grow immensely by the year 2050, meaning food production needs to increase by 70–100% to meet the future demand for food [5]. Furthermore, this poses a unique challenge to both producers and consumers. Additionally, there are several concerns associated with food insecurity: poor child development, transferrable diseases, mental illness, social disruption, suboptimal sleep patterns, and environmental sustainability [6].

### 1.1. Food Waste

The lessening of food waste plays a huge role in food security. Food loss and waste, as defined by the Food and Agriculture Organization (FAO), are associated with foods that are sold for consumption but end up getting wasted because of loss, waste, or other uses [7]. For example, food waste is associated with the loss of water and energy [8]. An Eastern European study found food waste and food loss were primarily predicted from issues associated with food harvesting [9]. Also, a Lithuanian study reported that households, retail, wholesale, and food services accounted for 70% of food waste [10]. With the growing population and millions of people suffering from undernourishment worldwide, the demand for global food production is steadily increasing [11]. Not only is food waste a problem at the consumer level, but it is also a major concern in the food production and post-harvest stages [12]. The unsustainable and wasteful behaviors of food products across supply chains have detrimental effects on society, as food waste exacerbates the challenge of food access and availability for consumers [13]. Food waste and climate change go hand in hand, and the impacts are catastrophic. The existing literature states that 95% of food waste ends up at landfill sites, where waste is then turned into carbon dioxide, methane, and other greenhouse gases [14]. Many factors contribute to food waste reduction. Additionally, the intent to minimize food waste stems from emotions of shame and guilt [15].

Rodgers et al. [16] stated that COVID-19 has positively impacted consumer behavior concerning food waste. Consumers have improved shopping habits and pushed toward a positive behavioral change regarding food waste, due to the loss of income opportunities during the COVID-19 lockdown [17]. In support of positive consumer behavior, [18] investigated young people’s knowledge of food waste and found this population had become more well informed regarding the environmental effects of food waste and had been encouraged to minimize waste. These changes in consumer behaviors are not the only area where the effects of COVID-19 can be observed. Notably, these behaviors were positive; on the other hand, the food supply side was severely affected by the COVID-19 lockdowns due to the closure of markets, restaurants, schools, bars, and hotels, making it challenging for consumers to access fresh foods [19]. Research indicates that as individuals grow older their attitudes about food waste become more worried, and women especially become more concerned about the negative effects of food waste [20]. With that said, there is a lack of research regarding women leaders’ efforts to mitigate food waste.

### 1.2. Women Leaders

Food security is rooted in each of the 17 United Nations Sustainable Development Goals (SDGs) and affects around 800 million people who lack access to food [6]. SDG 5, gender equality and women’s rights, is one of the 11 out of 17 goals that focus on gender equality [21]. SDG 5 is currently not on track to be achieved by the year 2030 due to the socioeconomic fallout from COVID-19 [22]. During the COVID-19 pandemic, female leaders were described as exuding excellent crisis leadership [23]. Furthermore, to successfully lead through crises requires a unique skill set. While focusing on leadership, fewer cases and deaths related to COVID-19 have been reported from countries with women as the head of state, and these countries are better prepared due to women’s preferences for public spending on healthcare [24]. Women global leaders responded to the pandemic in a more truthful, decisive, and empathetic manner compared to male leaders [25]. Research evaluating crisis leadership during COVID-19 found underrepresentation of female crisis leaders, which aligns with women being disproportionally represented within organizations’ leadership [26].

Studies in the United States have shown that there has been a rise in praise toward women leaders for having outstanding leadership abilities, and women more than men are associated with more effective leadership performance [27,28]. The role of women in agriculture needs to be modified to allow them to become more involved in decision making at both the professional and household levels [29]. The presence of women possessing leadership positions in the agricultural industry is growing [29]. To continue growing, women should associate with leadership mentors to strengthen their knowledge and networks, they should support one another, and they should envision themselves in both traditional and nontraditional roles as they pursue leadership positions in the agricultural industry [30].

### 1.3. Egocentric Networks

The authors in [31] define a network as ties between a set of actors. On the other hand, [32] delineates social networks as the structure and composition of relationships that link actors together. The relationships or ties among networks are critical components of the experience one has in a social setting, delineating the connections between individuals [33]. Analyzing the structure and composition of ties is essential in assessing the global balance of economic and political power [34]. Network research is often viewed as an analytical method; however, many researchers consider social networks as a theoretical perspective with opportunities for a variety of analyses [35]. In other words, behavior is based on social interaction, rather than beliefs, individual motivation, or calculation [34]. Egocentric research, as opposed to socio-centric research, focuses on individuals and their personal networks. A network study conducted by [36] discusses the disadvantages associated with dense networks, i.e., those with more strong ties, among a group of 700 Chinese entrepreneurs. Another study investigated why women have less success with networking and found that the existence of structural barriers in the form of homophily and work–family hamper women’s networks [37]. Strong ties refer to those when an individual shares common opportunities and knowledge; while weak ties are not important to an individual but are essential in sharing new information and access to other social systems [34]. The investigation of these ties and the strength of relationships among women leading in agriculture’s personal networks is what researchers sought to examine in this study.

## 2. Theoretical Framework

Over the past few decades, several crises have increased the opportunity for researchers to explore crisis leadership [17]. A crisis is a rare public situation that causes detrimental outcomes for numerous individuals, including businesses and their stakeholders, requiring successful leadership [21]. Crises vary among individuals and circumstances. When organization leaders adopt a learning orientation, crises are predicted to be perceived as opportunities [38]. The Farm Bureau supplies its leaders and members with sufficient opportunities for professional development for leaders to become more effective during crisis events. A leader’s ability to learn and reflect is critical for the success of an organization [39]. With that said, this study explored a crisis leadership framework to explore the characteristics of women leading in agriculture and their networks during COVID-19.

### Crisis Leadership

In the past few decades, we have experienced several crises including the Chornobyl nuclear disaster, the tsunami in the Pacific region, the Asian Financial Crisis, the 2008 Global Economic Crisis, the Sichuan earthquake, the Eurozone Debt Crisis, and COVID-19 [40]. The context of a crisis can manipulate leadership behaviors [41], along with the personal characteristics, mindsets, and actions of leaders, which can severely impact the internal and external stakeholders of organizations [42]. Research revolving around crisis leadership remains fragmented, making it difficult to fully understand the current state of the field [40]. As defined by [43], a crisis is a process that weakens the normal functioning of an individual, organization, or community. When crises are well managed, there is an opportunity for positive turning points [44]. For example, COVID-19 severely affected the everyday functioning of organizations and individuals’ lives, causing a decline in revenue [45]. On the other hand, the pandemic provided new ventures for organizations to prove their adaptability [46].

COVID-19 has altered the way individuals think and react. Researchers are in a unique position to study the leadership capacity and competencies of individuals during this ongoing crisis [47,48]. Some critical leadership competencies during COVID-19 include communication, quick incident preparation, proactivity, quick implementation, and both optimistic and realistic attitudes [49].

Previous studies emphasized four critical crisis leadership competencies: a sense of urgency, strong emotional intelligence, problem-solving skills, and communication [50,51]. A leader’s sense of urgency is crucial for recognizing a crisis and developing an action plan [50]. Factors including personal risk perceptions can affect a leader’s sense of urgency [52]. With more complex risks, like climate change, a sense of urgency can also be relevant [53]. For extended crises, a problem exists as prolonged time periods diminish leaders’ sense of urgency [54].

The next crisis leadership competency, leaders’ emotional intelligence, plays a vital role during crisis events [55]. Flexibility, the ability to adapt to change, and moderating emotions to encourage positive emotional responses from followers are factors that contribute to a leader’s emotional intelligence [56].

Problem solving, the next crisis leadership competency, refers to an analytical mindset possessed by leaders to respond to problems and prevent reoccurring damage [57].

The final leadership competency, communication, is the most crucial competency both before and during a crisis event [58]. Communication is directly associated with the speed of decision making and action, along with the scrutiny and publicity of crisis management efforts [59]. Researchers suggest the development of skills including negotiation, management, delegation, and relationship building during crises [60,61]. Along with crises are high levels of risk associated with immediate and continued loss [62], requiring organization leaders to be proactive in preparing for potential critical events [1]. In times of crisis, researchers who investigated the differences in trust between men and women leaders who acquire relational behaviors found women had a unique advantage [50].

Currently, there is a gap in the literature concerning women leaders in agriculture and their influence regarding food waste. Therefore, the purpose of this research was to explore the factors associated with women in agricultural leadership leading their personal network members to positive food waste decisions during COVID-19. Understanding women leading in agriculture’s personal advice networks allows researchers to investigate the influence of their leadership. The two research objectives that guided this study were the following:

1. Describe the personal advice networks of women involved in the Farm Bureau’s women’s leadership committees.
2. Examine the individual, dyadic, and network-level factors related to Women’s Leadership Committee members connecting with people they lead to positive food waste behaviors.

This manuscript is structured in a way that introduces the purpose of this research, followed by a detailed section on the research design and study implementation. In the Materials and Methods section, details are provided as to why we had to disseminate two surveys. Additionally, the participants’ personal characteristics are described. Furthermore, the variables are outlined, along with how the data were analyzed using our unique study design. Following the section on methods, the results are presented. Lastly, the manuscript ends with Discussion and Conclusions sections to provide a better understanding of the results of this novel study.

## 3. Materials and Methods

In this study, we conducted a mixed-methods egocentric network analysis of women involved in one of the southern region Farm Bureau’s women’s leadership programs to assess advice networks during COVID-19. Originally, a survey was sent to the 12 southern region states, where we received responses from 50 women (*n* = 50) or “egos”. The southern region states were Alabama, Arkansas, Georgia, Florida, Kentucky, Louisiana, Mississippi, North Carolina, Oklahoma, South Carolina, Tennessee, and Virginia. There were approximately 159 (*N* = 159) total women among the 12 southern region states’ women’s leadership committees. Upon analyzing the data and determining an unreliable construct (opinion leadership), we chose to send the survey with a revised construct to the remaining states who had active women’s leadership programs in the United States. A second survey was administered to the remaining 19 states who had an active women’s leadership program, with a revised opinion leadership construct. The 19 states were Arizona, Colorado, Connecticut, Delaware, Indiana, Kansas, Maryland, Montana, Nevada, New Hampshire, New Jersey, New Mexico, Oregon, Pennsylvania, Rhode Island, South Dakota, Utah, Washington, and West Virginia. There were 32 (*n* = 32) responses to the second survey (national survey), out of approximately 170 (*N* = 170) total women among the 19 remaining states’ women’s leadership committees. Of the 50 responses from the southern survey and 32 from the national survey, the participants identified 410 network members, or “alters” as we will refer to them throughout this manuscript.

Researchers chose to conduct an egocentric network analysis to better understand the social relationships and influences that these women sought advice from since COVID-19 began. Throughout this paper, we refer to participating women as “egos” and the individuals in their social networks as “alters” [62]. Egocentric data, also commonly referred to as local or personal network data, consists in asking questions in which individuals’ responses provide relational information to better understand their personal network characteristics and their influence on behavior [63]. Egocentric network research is achieved by asking respondents (i.e., egos) to elicit members of their social system (i.e., alters) and then collecting information on their nominated alters based on the egos’ knowledge and perception and investigating the ties between them [32,64].

### 3.1. Participants and Procedure

The study sample was comprised of women holding leadership positions within one of the 32 United States’ Farm Bureau’s women’s leadership committees. Therefore, this study is representative of women who possess leadership positions among their states’ Farm Bureau’s women’s leadership committee. Additionally, the results allowed us to explore ego and alter relationships to better understand the food waste behaviors of women leading in the Farm Bureau. To recruit all members from the leadership program, a list of all leadership coordinators from each of the Farm Bureaus was obtained and contacted to gain approval to survey the women’s leadership committees. All 32 of the states’ Farm Bureau leadership coordinators approved of this study. Upon approval, researchers developed the instrument and shared the link with the leadership coordinators to share with the committees. This was chosen as the delivery method due to the confidentiality of the women’s personal information (i.e., email), and leadership coordinators felt the survey would produce a higher response rate if they encouraged their committees to participate.

A cross-sectional survey was constructed and distributed using Qualtrics to collect qualitative and quantitative data. According to [65], a cross-sectional survey is when information is collected at one point in time, although the time it takes to collect data may range from a day to a couple of weeks. The southern and national surveys were identical, except for the opinion leadership assessment. The surveys were designed to measure ego level, alter level, network level, and personal characteristics.

### 3.2. Variables

The ego-level (i.e., individual-level) questions included a series of demographic questions, a seven-item crisis leadership assessment, and a three-item COVID-19 perception assessment. The demographic questions were formatted as both short response and multiple choice. The short-response questions included the participants’ state women’s leadership committee, their position held with the committee, and their birth year. The multiple-choice questions asked participants to select how long they have been involved in the women’s leadership program, what race or ethnicity they identify with, and their highest level of education. The items within the two assessments are described in the tables below, accompanied by the mean and standard deviation (see Table 1 and Table 2).

The table below depicts the results of the three assessments egos took for the southern survey. There were eight items in the food waste behavior assessment. Positive scores were associated with *I learned about food waste* (*M* = 0.40, *SD* = 1.10), *I reflected on my food waste management* (*M* = 0.40, *SD* = 0.99), *I assisted with food waste damage control* (*M* = 0.34, *SD* = 1.11), and *I treated the food waste cause* (*M* = 0.11, *SD* = 1.13) and indicate positive food waste behavior during COVID-19. The last three items were associated with negative scores (*I established norms for divergent thinking regarding food waste* (*M* = −0.11, *SD* = 1.07), *I emphasized short and long-term food waste outcomes* (*M* = −0.13, *SD* = 1.06), and *I sought views of multiple stakeholders regarding food waste* (*M* = −0.33, *SD* = 1.06)), meaning egos were likely not performing these behaviors. Three items assisted with measuring egos’ perception of COVID-19. All items were positive: *change* (*M* = 0.36, *SD* = 1.14), *innovation* (*M* = 0.33, *SD* = 1.06), *reputation enhancement* (*M* = 0.14, *SD* = 1.15), indicating that egos view COVID-19 as an opportunity (see Table 1).

The table below depicts the results from the two assessments that egos took for the national survey, with the revised opinion leadership construct. There were eight items in the food waste behavior assessment. Positive scores were associated with *I reflected on my food waste management* (*M* = 0.48, *SD* = 1.12), *I assisted with food waste damage control* (*M* = 0.27, *SD* = 1.26), *I treated the food waste cause* (*M* = 0.27, *SD* = 1.26), and *I learned about food waste* (*M* = 0.03, *SD* = 1.20) and indicate a positive food waste behavior during COVID-19. The last three items were associated with negative scores (*I established norms for divergent thinking regarding food waste* (*M* = −0.07, *SD* = 1.23), *I emphasized short and long-term food waste outcomes* (*M* = −0.10, *SD* = 1.19), and *I sought views of multiple stakeholders regarding food waste* (*M* = −0.30, *SD* = 1.24)), meaning egos were likely not performing these behaviors. Three items assisted with measuring egos’ perception of COVID-19. All items were positive: *change* (*M* = 0.87, *SD* = 1.01), *innovation* (*M* = 0.70, *SD* = 1.06), and *reputation enhancement* (*M* = 0.57, *SD* = 1.14), indicating that egos view COVID-19 as an opportunity.

Egos were asked three types of questions to successfully complete the egocentric network analysis: a name generator, name interpreters, and alter–alter ties. Name generators are questions that elicit a list of alters with whom the ego is connected to in a specific way, name interpreters assess characteristics of the nominated alters, and alter–alter ties measure the structure present within the personal network [35]. Alter-level variables were determined based on egos’ responses to the chosen name generator which asked participants to “please provide the initials of the 5 people you would go to for advice.” The type of name generator that was asked can be referred to as an affect-based name generator, where participants nominate individuals that they are close to and have a strong relationship with [35]. There were 10 name interpreters included in this study. The first four questions asked the ego to select the type of relationship, gender, how often they communicate, and how long they have known each alter. Researchers chose the last six questions to align with the research objectives and theoretical framework using five-point Likert scales (−2 = *never*, −1 = *rarely*, 0 = *sometimes*, 1 = *usually*, 2 = *always*), except for one short-response question. Two questions assessed opinion leadership, with the first asking if the alter would describe the ego as an opinion leader and the second asking if the ego would describe the alter as an opinion leader. It should be noted that the definition of opinion leader was provided in the survey in case the egos were unfamiliar. The following two short-response questions were about food waste leadership and influence, with the first asking egos if they lead the alter into making positive food waste decisions and the second asking egos to describe how the alter influences their food waste decisions. The final two name interpreters asked about trust, one asking if the egos trust the alter, and the other asking if the alter would describe the ego as trustworthy.

For this study, researchers analyzed ties between alters. This is a critical measure because alter-alter ties form the basis of structural measures for ego networks [35]. To accurately measure alter–alter ties, the ego was asked a series of questions stating “does (alter 1–5) know alter (1–5)?” until all ties were accounted for.

To compute all network-level variables, E-Net statistical software .032 was used [64]. To assess the composition and structure, three kinds of data were needed: the number and nature of relationships between egos and alters, the characteristics of alters, and the pattern of relationships among alters [35], all derived from the name generator, name interpreter, and alter–alter tie questions.

The following aggregate network-level measures were assessed for the southern and national surveys: network size (total number of alters in each ego network), heterogeneity (the range of network relationships, communication, length known, food waste leadership, trust, and opinion leadership), density (degree of connectedness of alters), structural holes (absence of tie between two alters), effective size (number of nonredundant alters that ego is connected to), constraint (the extent to which ego is connected to few others), and the proportion (average percentage of a network that possesses certain characteristics or feelings) of the network that was known for five or more years, trusts ego, considered trustworthy, female, described as an opinion leader, would describe the ego as an opinion leader, family, and communicates with ego every day.

### 3.3. Data Analysis

A descriptive analysis took place for ego- and alter-level variables. Frequencies and percentages were calculated for the southern and national surveys’ ego and alter demographic information including alter gender, alter relationship, alter communication, alter length known, ego state, ego committee position, ego duration of involvement in women’s leadership program, ego generation, ego race/ethnicity, and ego education. Means and standard deviations were determined for the following ego and alter descriptive data: ego trust in alter, alter trust in ego, ego describes alter as opinion leader, alter describes ego as opinion leader, ego leads alter toward positive food waste decision, ego-level food waste behavior assessment, ego-level crisis leadership assessment, ego-level opinion leadership assessment.

E-Net statistical software, identified by Borgatti, was used to assist the researchers with analyzing the composition and structure of ego networks [64]. The composition, heterogeneity, and structural holes measured were computed for each survey. Network variables were used as predictor variables in the multiple linear regressions.

To identify factors related to egos leading alters toward positive food waste decisions, we examined a multilevel model computed using the multilevel package [65] within R 4.3. programming language and software [66]. Multilevel modeling is an ideal analytic strategy when conducting egocentric network analyses due to its ability to account for the variance between and within ego networks [54,67]. Based on intraclass correlation calculations and likelihood ratio tests, we determined a random coefficient model was most appropriate for our data [68,69]. A random coefficient model adjusts for a lack of independence between observations of nominated alters by including a random intercept for each ego, as well as an adjusted slope for each ego based on whether an alter had experienced sexual abuse [35]. Independent variables included in the random coefficient model were ego’s generation (millennial, Generation X, and baby boomer), race, crisis leadership sum score, and food waste sum score; alter relationship (professional, friend, pastor, neighbor), race, gender, and Farm Bureau membership; length of relationship between alter and ego and frequency of communication between alter and ego; if ego considered alter to be an opinion leader and if alter considered ego to be an opinion leader; if alter trusts ego; proportion of the network that describes ego as an opinion leader “always”, that ego describes as an opinion leader “always”, that communicates with ego daily and is female; and network density. We conducted a test of multicollinearity and determined that none of the independent variables were concerning, due to variance inflation factor (VIF) scores ranging from 1.04 to 2.12 for all variables.

The surveys were composed of one open-ended question to describe alters’ (105) influence on egos’ food waste behavior. The answers to this question were analyzed to determine themes among responses. Data were analyzed through the lens of a woman in agriculture who was familiar with the population. The qualitative data for each survey were put into an Excel spreadsheet where the researcher read through each response and identified themes that emerged.

For the southern survey, there was a total of 117 responses out of the 250 possible, which were categorized into five themes: conservation practices, alter and ego discussion, alter money consciousness, alter experience, and no influence. For the national survey, there was a total of 101 responses out of the 160 possible, which were categorized into six themes: no influence, conservation practices, alter leads by example, alter and ego discussion, alter and ego system development, alter money consciousness, and guilt. The themes are described further in the results section.

### 3.4. Validity and Reliability

For this study, a panel of experts reviewed the survey to assess validity [70]. The research committee was composed of knowledgeable researchers with quantitative and qualitative backgrounds along with social network analysis expertise. For qualitative data, themes were identified for the short-response questions and reviewed by the research committee to prevent data collector bias and to determine validity.

The internal reliability of the instrument was determined by post hoc Cronbach’s alpha scores [68,71]. According to [69,72], reliability coefficients larger than 0.70 are considered reliable. Cronbach’s alpha scores were determined for the three assessments within the southern survey instrument: food waste leadership construct (α = 0.92, 7 items), crisis leadership construct (α = 0.96, 3 items), and opinion leadership characteristics construct (α = 0.74, 6 items). Researchers decided to remove the item that produced the lowest reliability score for the opinion leadership construct. The item that was removed was “greater exposure to diverse media.” Upon removing the item, the [68] alpha score increased (α = 0.76, 5 items). For the national survey, the Cronbach’s alpha scores were determined for the food waste behavior construct (α = 92, 7 items), crisis leadership construct (α = 94, 3 items), and opinion leadership construct (α = 83, 7 items). Researchers removed the “I conform to Farm Bureau norms” item from the opinion leadership construct to produce a higher reliability score.

## 4. Results

Objective one sought to describe personal advice networks. Starting with understanding the egos, Table 3 presents the demographic information we collected through our surveys. Egos were from all over the United States, with a larger number of egos from Louisiana (*n* = 9, 11%), Florida (*n* = 9, 11%), Indiana (*n* = 8, 9.8%), Oklahoma (*n* = 7, 8.5%), and South Carolina (*n* = 5, 6.1%). The majority of egos held district representative (*n* = 29, 35.4%) or member (*n* = 23, 28%) positions on their states’ committee. Egos mostly indicated being involved in their women’s leadership committee for five or more years (*n* = 36, 43.9%) or six months to a year (*n* = 10, 12.2%). A little more than half of egos were part of the baby boomer generation (*n* = 42, 51.2%). Close to 100% of egos indicated being White or Caucasian (*n* = 75, 91.5). Most of the egos were educated and possessed a bachelor’s (*n* = 31, 37.8%) or master’s (*n* = 19, 23.2%) degree.

In Table 4, the alter demographic information is displayed. There was a total of 410 alters identified by egos. A majority of alters were female (*n* = 256, 62.4%). Most of the alters were either family members (*n* = 186, 45.4) or friends (*n* = 94, 22.9%). Communication varies among alter and egos with a higher number of alters and egos who communicate every day (*n* = 99, 24.1%), every few days (*n* = 82, 20%), or every week (*n* = 70, 17.1%). More than half of alters have known their ego for five or more years (*n* = 352, 85.9%).

Objective 2 sought to examine the individual, dyadic, and network-level factors related to Women’s Leadership Committee members connecting with people they lead to positive food waste behaviors. Women were more likely to report connections with people they led to positive food waste behaviors when they had higher food waste sum scores (*b* = 0.08, *p* < 0.001), they were part of Generation X (*b* = 1.92, *p* = 0.04), the alter they led to more positive food waste behaviors was a friend (*b* = 0.37, *p* = 0.03), and if there were fewer women in their advice networks (*b* = −0.01, *p* = 0.04) (see Table 5).

Table 6 presents the statements from the qualitative question according to theme. The qualitative question in our survey sought to understand the influence of alters on egos’ food waster behavior. Upon analyzing the responses, eight themes emerged: no influence, conservation practices, alter and ego discussion, alter leads by example, alter money consciousness, alter experience, alter and ego system development, and guilt. There was a total of 159 responses. Most egos indicated not being influenced by alters (*n* = 59). Additionally, there were 46 egos who were influenced by conservation practices mentioned by an alter.

There were 54 responses removed from coding as they did not answer our qualitative question, and they are organized below (see Table 7). Most of the responses removed were one word: *no* (*n* = 27, 50%), *somewhat* (*n* = 10, 18.51%), or *yes* (*n* = 6, 11.11%). After a peer debrief, we decided to remove the following responses due to their lack of clarity.

## 5. Discussion

This study promoted a deeper understanding of the advice networks of women leading in agriculture to improve food security. Importantly, this analysis showed us that when women did lead people to positive food waste behaviors, it was when they themselves had high food waste sum scores, were from Generation X, if their alter was a friend, and if their networks had fewer women.

The research in [73] on networks in the workplace indicates that the stronger the ties within a network, the less exposure to new information. Due to the density of their networks, Farm Bureau women’s committee members are limited to few information sources regarding food waste, supporting the findings in [73] that having multiple sources of input within their networks was related to important leadership characteristics. The less dense networks may be helpful with supplying egos with more diverse thoughts in regard to leadership and food waste. This finding is concerning, and more research should be conducted to examine the correlation between the density of networks and women leading in agriculture. Women were described as excellent leaders through COVID-19 [41] with more truthful, decisive, and empathetic competencies [43]. The findings in this study indicate Generation X women with higher food waste sum scores and networks with fewer women were leading their friends toward positive food waste [8]. It is likely that gender-related barriers among the Farm Bureau organization are contributing to women’s leadership success [74], implying that members of women’s personal networks could be more influenced from male leaders regarding food waste. It is likely that due to the chosen name generator, the advice network members had more influence on egos’ food waste behaviors. Our qualitative data indicate the variety of ways alters have influence over egos’ food waste behaviors. In the attempt to determine how alters influence egos’ food waste decisions, there were several statements regarding sharing food with animals, which was consistent with prior research [75].

The authors in [26] discussed proactivity, critical incident preparation, quick implementation, communication, and a realistic optimistic attitude as necessary competencies for leaders during COVID-19. However, women in this study failed to identify characteristics and crisis leadership perceptions similar to those addressed by [26]. Previous research found positive food waste management and purchasing habits among consumers during COVID-19 [14]. The environmental effects of food waste have become more concerning to young people during COVID-19, encouraging food waste minimization [15]. With the majority of women in this study being baby boomers, it can be inferred that those of an older generation are less concerned with environmental effects. The existing literature indicates a need for more food waste communication to minimize food waste impacts to achieve food security [76] and to meet the SDGs [40]. Developing a relationship and collaboration between the Farm Bureau and agricultural extension systems may assist with meeting the SDGs and achieving food security. Researchers found a lack of engagement between the two entities that upon collaboration would provide a more well-rounded and diverse group of agricultural leaders. This study emphasized the need for more communication among women and their social systems regarding food waste management and the impact associated with failing to create improved food disposal practices. Women have been cited as better caretakers for the environment [39], making them essential leaders during catastrophic events. The women involved in this study are leaders within their local communities and state who advocate for agriculture and work to implement American Farm Bureau priority issues. Yet, regarding food waste, women were not leading their network peers and did not view COVID-19 as a time for reflecting, learning, divergent thinking, goal setting, treating, or assisting with food waste damage control. It is likely that women are not leading food waste change during crisis events and therefore missing opportunities to improve local food security.

The ability for agricultural research institutions to form relationships with farm organizations allows for the unique opportunity to collaborate on various projects. Agricultural research institutions conduct studies on areas of the agricultural industry and present those findings in a variety of outlets, whether that be in various journal articles, university presentations, or international/national conferences. Extension agents play a role in the dissemination of research findings among the industry to improve production, leadership, or communication practices. Therefore, more collaboration between farm organizations and agricultural research institutions is necessary to improve leadership competencies and practices regarding food waste. Overall, more research needs to be conducted on women leading in agriculture, specifically regarding food waste management and crisis leadership competencies.

It is recommended that practitioners promote new relationships and collaborations to expand women’s networks, ultimately leading to more knowledge and other social networks with various perspectives and experiences. The Farm Bureau is a homogeneous group. Committee members and leadership coordinators need to cultivate a plan to recruit more diverse populations, to build a community of diverse women leaders in agriculture to share their stories and positively influence the industry [77]. More women are needed to fulfill agricultural leadership roles to make a positive global impact on improving the sustainability, safety, and security of our food system [50]. Change agents from states’ Farm Bureaus should contemplate prioritizing community building, communication, and development of opinion leaders [78,79] to assist with achieving food security. Farm organizations need to execute a strategy to develop their members’ food waste competencies and leadership, so that women involved in the committee can confidently and positively influence their network peers.

This study’s limitations include that it is composed of self-reported data. The research was not generalizable to the whole population, only to the network of the women committee members from the Farm Bureau’s women’s leadership programs. Another limitation of this study was researcher bias because the researcher had prior experience conducting research on a female Farm Bureau population. There was a limitation concerning egocentric network analysis regarding participants’ ability to enumerate the most appropriate ties related to the chosen name generator. Another limitation was the nomination limit of five which provided only a small glimpse into women’s personal advice networks. In this study, the small sample size was a limitation. The sample was only comprised of a small number of women involved in committees compared to the total number of women involved. The sample only included those involved in the Farm Bureau’s women’s leadership programs; therefore, the results indicated a narrow perspective of women leading in agriculture. Due to the research being conducted on affect-based networks, there was a limitation based on the limited network size and range due to a more intimate name generator [54].

## 6. Conclusions

To address sustainable development goal (SDG) 1 (no poverty), SDG 2 (zero hunger), and SDG 11 (sustainable communities), researchers and practitioners need systems thinking approaches to develop solutions for the interdisciplinary and multi-dimensional nature of food systems. The variables that impact food waste and food sustainability go beyond the scholarship presented here. However, our neoteric paradigm investigation with women agricultural leaders illuminated future study pathways for interdisciplinary scholars to develop solutions that simultaneously improve science and practice for food systems actors under the auspices of the SDGs.

The research we conducted uniquely examines the personal advice networks of women leading in agriculture and their influence on food waste behaviors. This novel approach provides a perspective on how interpersonal relationships and networks within the realm of agriculture impact individual food waste decisions, particularly during COVID-19. While there is a plethora of research on leadership in agriculture and food waste behaviors, this study specifically targets women in agricultural leadership positions. Additionally, this research aligns with current global challenges and underscores the timeliness and relevance of the research.

Our inquiry advances crisis leadership theory through the investigation of competencies: a sense of urgency, strong emotional intelligence, problem-solving skills, and communication. The sense of urgency, from this study, was highlighted from women leaders’ role in improving food waste during and after COVID-19. Our findings agreed with the literature that women have higher emotional intelligence and are therefore apt to be more effective as crisis leaders in food waste paradigms. The women were well positioned to share and execute problem-solving skills to their networks. Women and their networks acted as opinion leaders diffusing food waste practices. Lastly, our inquiry advances crisis leadership’s specialized theory through the lens of food waste and its interdisciplinary realm juxtaposed to natural disasters often studied by using crisis leadership to scaffold their studies. The alignment of SDGs 1 (no poverty), 2 (zero hunger), and 12 (sustainable consumption and production patterns) also advances crisis leadership theory on a more global scale.

Women are underrepresented in leading food waste and food loss initiatives. Though the reasons are void in the literature and not revealed from our study, more efforts need to be made to investigate women’s perspectives, decisions, and capacity to disseminate food waste innovations, programs, information, or technologies to members of their respective social systems. These scholarly additions would enhance the understanding of food waste behavior’s social implications, particularly from women’s lenses. Scholars should examine dissimilar human dimension indicators of food waste and food loss [9,10] to advance the inquiry beyond our study and potentially develop solutions that improve these issues, which would simultaneously address SDG 1, SDG 2, and SDG 12.

## Figures and Tables

**Table 1:** Ego Self-Assessment Results for Southern Region Survey.

Variables	*n*	*M*	*SD*
During COVID-19			
I learned about food waste	47	0.40	1.10
I reflected on my food waste management	47	0.40	0.99
I assisted with food waste damage control	47	0.34	1.11
I treated the food waste cause	47	0.11	1.13
I established norms for divergent thinking regarding food waste	47	−0.11	1.07
I emphasized short and long-term food waste outcomes	47	−0.13	1.06
I sought views of multiple stakeholders regarding food waste	46	−0.33	1.06
I view COVID-19 as an opportunity for food waste			
Innovation	48	0.33	1.06
Change	44	0.36	1.14
Reputation Enhancement	43	0.14	1.15

Note. −2 = Strongly Disagree, 2 = Disagree, 0 = Neither Agree nor Disagree, 1 = Agree, 2 = Strongly Agree.

**Table 2:** Ego Self-Assessment Results for National Survey.

Variables	*n*	*M*	*SD*
During COVID-19			
I reflected on my food waste management	31	0.48	1.12
I learned about food waste	31	0.03	1.20
I assisted with food waste damage control	30	0.27	1.26
I treated the food waste cause	30	0.27	1.26
I established norms for divergent thinking regarding food waste	30	−0.07	1.23
I emphasized short and long-term food waste outcomes	30	−0.10	1.19
I sought views of multiple stakeholders regarding food waste	30	−0.30	1.24
I view COVID-19 as an opportunity for food waste			
Change	30	0.87	1.01
Innovation	30	0.70	1.06
Reputation Enhancement	30	0.57	1.14

Note. −2 = Strongly Disagree, 2 = Disagree, 0 = Neither Agree nor Disagree, 1 = Agree, 2 = Strongly Agree.

**Table 3:** Ego Descriptive Results for Southern Region and National Surveys (*n* = 82).

Variables	*n*	%
Ego State		
Louisiana	9	11
Florida	9	11
Indiana	8	9.8
Oklahoma	7	8.5
South Carolina	5	6.1
Utah	4	4.9
Alabama	4	4.9
Arkansas	4	4.9
Tennessee	4	4.9
Arizona	3	3.7
New Mexico	3	3.7
Colorado	3	3.7
Mississippi	3	3.7
Montana	3	3.7
Rhode Island	2	2.4
Georgia	2	2.4
North Caroline	2	2.4
Kansas	2	2.4
Nevada	2	2.4
West Virginia	1	1.2
Virginia	1	1.2
Maryland	1	1.2
Ego Committee Position		
District Representative	29	35.4
Committee Member	23	28
Chair	14	17.1
Vice Chair	10	12.2
Secretary	3	3.7
Ego Involvement in Women’s Leadership Committee		
5 or more years	36	43.9
6 months to 1 year	10	12.2
1 to 2 years	8	9.8
3 to 4 years	8	9.8
2 to 3 years	7	8.5
4 to 5 years	7	8.5
Less than 6 months	3	3.7
Ego Generation		
Baby Boomer	42	51.2
Generation X	19	23.2
Millennial	14	17.1
Silent	1	1.2
Ego Race/Ethnicity		
White or Caucasian	75	91.5
American Indian or Alaska Native	3	3.7
European American	1	1.2
Biracial *	1	1.2
Ego Education		
Bachelor’s	31	37.8
Master’s	19	23.2
Some College	17	20.7
Associate’s	7	8.5
Professional Degree	4	4.9
High School Diploma	2	2.4

Note. * The biracial category indicates someone identifying as White or Caucasian and American Indian or Alaska Native.

**Table 4:** Alter Descriptive Results for Southern Survey and National Surveys (*n* = 410).

Variables	*n*	%
Alter Gender	410	
Female	256	62.4
Male	146	35.6
Alter Relationship	410	
Family	186	45.4
Friend	94	22.9
Farm Bureau	91	22.2
Professional	25	5.9
Pastor	3	0.7
Neighbor	2	0.5
Rancher	1	0.2
Alter Communication	410	
Every Day	99	24.1
Every Few Days	82	20.0
Every Week	70	17.1
Every Few Weeks	68	16.6
Every Few Months	42	10.2
Every Month	25	6.1
1 to 2 Times Per Year	15	3.7
Less than Once Per Year	1	0.2
Alter Length Known	410	
5 or More Years	352	85.9
4 to Almost 5 Years	17	4.1
3 to Almost 4 Years	11	2.7
1 to Almost 2 Years	8	2.0
2 to Almost 3 Years	6	1.5
6 Months to 1 Years	5	1.2
Less than 6 Months	2	0.5

**Table 5:** Multilevel model.

Variables	*b*	*t*	*p*
Intercept	−1.50	−0.91	0.36
Ego Millennial	1.92	2.07	0.04 *
Ego Generation X	1.55	1.67	0.08
Ego Baby Boomer	1.55	1.76	0.10
Alter Professional Relationship	0.39	1.29	0.20
Alter Friend	0.37	2.16	0.03 *
Alter European American	0.33	0.37	0.71
Alter Neighbor	0.30	0.28	0.78
Alter Pastor	0.28	0.45	0.65
Alter Farm Bureau	0.25	1.29	0.20
Ego Biracial	0.13	.15	0.88
Ego Food Waste Sum Score	0.08	3.95	0.00 *
Alter and Ego Length Known Each Other	0.06	.98	0.32
Alter Opinion Leader	0.06	.66	0.51
Proportion of Network Describe Ego Opinion Leader Always	0.01	1.35	0.18
Proportion of Network Communicate with Ego Daily	0.00	0.71	0.48
Network Density	−0.30	−0.13	0.90
Ego White or Caucasian	−0.16	−0.34	0.74
Alter Gender	−0.13	−0.92	0.36
Alter Describe Ego Opinion Leader	−0.04	−0.38	0.70
Alter and Ego Communication	−0.03	−0.72	0.47
Proportion of Network Female	−0.01	−2.13	0.04 *
Alter Trusted by Ego	−0.01	−0.07	0.94
Proportion of Network Ego Describe as Opinion Leader Always	−0.00	−0.72	0.48
Ego Crisis Leadership Sum Score	−0.00	−0.06	0.95

Note. * *p* < 0.05.

**Table 6:** Alter Food Waste Influence Statements for Southern Survey (*n* = 159).

Theme	Example	*f*
No Influence		59
	“He/she doesn’t.”	32
	“Not at all.”	13
	“Not in our discussion.”	11
	“Never.”	2
	“We have never discussed food waste that I can recall.”	1
	“Negatively.”	1
	“Only discuss the issue.”	1
	“Not a subject with our friendship.”	1
	“I don’t feel that he does.”	1
	“We don’t see each other often enough to instill food waste decisions.”	1
	“Doesn’t usually, as he doesn’t know much about ways to prevent.”	1
Conservation Practices		46
	“We donate extra.”	4
	“Utilizing what is on hand.”	3
	“Being conservative.”	3
	“Leftovers, have it for later.”	2
	“She supports my compost program.”	2
	“Utilizing leftovers.”	2
	“She and I talk about ways to preserve food.”	1
	“Eat leftovers.”	1
	“Utilizes compost containers.”	1
	“Always feed leftovers to the animals.”	1
	“Encourages me to not cook too much and eat leftovers.”	1
	“Don’t throw extra food away.”	1
	“Shares common practices in food preservation.”	1
	“Eats a lot.”	1
	“We share leftovers.”	1
	“Uses leftovers for a family meal or into soups and casseroles.”	1
	“Leftover food is always shared with others or animals.”	1
	“She helps me save items for compost.”	1
	“Shop small and utilize spices for variety of flavor.”	1
	“We discuss recipes for using leftovers.”	1
	“Plan a weekly menu”.	1
	“Aid like the Little Red Hen. She gathers her food even if she already has some in supply. She sets herself up to share with others what she has gathered.”	1
	“Buy bulk, and store in a large freezer with “use by” labels.”	1
	“Freezing leftovers.”	1
	“What to do with extra food.”	1
	“Highly. She has been the one to teach me how to persevere and use what we have.”	1
	“She is a good example of saving and food storage.”	1
	“She has taught me how to persevere and reuse things I need to.”	1
	“Using leftovers.”	1
	“Planned shopping, freezing leftovers, quantity discounts, using food storage.”	1
	“She taught me friendly useful habits.”	1
	“Planned shopping.”	1
	“He is willing to eat leftovers and is good at making sure we utilize the food we have in the best way.”	1
	“She is good at taking only what she will eat.”	1
	“Brings up ways to repurpose food, reminds us to not let it go to waste and to finish everything.”	1
	“She has chickens and convinces me to give her my waste.”	1
Alter and Ego Discussion		19
	“Open discussions.”	5
	“He/she discusses the importance.”	4
	“Reminds me if wasteful.”	1
	“Shares information from other regions.”	1
	“Makes me aware of the sustainability aspect of our food.”	1
	“Through education.”	1
	“By Challenging my thought process about food waste.”	1
	“Appoints out our responsibility to consumers about food safety and availability.”	1
	“We discuss food security in communities.”	1
	“Discussion of school situations.”	1
	“Shares the importance of the production of our food and the need to see the food supply chain is functional for all citizens.”	1
	“We share gardening advice.”	1
	“Shares information.”	3
	“By sharing credible information.”	1
	“Shares new ideas that she has tried.”	1
	“He encourages me to be resourceful.”	1
	“Listened to webinars and have shared ideas with me how to conserve food.”	1
	“By discussing food and animals in a classroom setting for children.”	1
	“I listen to her.”	1
	“Through discussion.”	1
Alter Lead by Example		10
	“Opinion and by example.”	7
	“She is a leader in them.”	1
	“He is the one that inspired our company’s food waste decision.”	1
	“She never wastes food and has instilled the same principles in me.”	1
Alter Money Consciousness		5
	“Food costs money. Wasting food wastes money.”	1
	“He taught me to be frugal.”	1
	“She is a home economist and I consult with her and seek her ideas.”	1
	“She is very health-conscious and frugal.”	1
	“Discuss the costs and portions of food services and she shares ways to cut costs.”	1
	“Budgeting expenses.”	1
	“Mostly by reminding me of the cost associated with food waste.”	1
Alter Experience		4
	“His career experiences contribute to my judgment on food waste.”	1
	“Her medical background gives me a broader knowledge concerning food waste decisions.”	1
	“His broad experience in the agriculture and social setting can influence my food waste decisions.”	1
	“As our parish president, his advice and opinions greatly influence my food waste decisions.”	1
Alter and Ego System Development		3
	“We live in the same household so we have a system that works for us.”	2
	“By familial habits.”	1
Guilt		1
	“Guilt.”	1

**Table 7:** Qualitative Statements Removed from Coding for Southern Survey (*n* = 54).

Example	*f*	%
“No”	27	50.00
“Somewhat”	10	18.51
“Yes”	6	11.11
“Rarely”	4	0.74
“Sometimes”	3	0.56
“Little influence”	1	0.19
“Verbal”	1	0.19
“Cooperating”	1	0.19
“Uncertain”	1	0.19
“I make my own.”	1	0.19
“Not much”	1	0.19
“I feel there is too much food eaten in today’s society that is prepared and packaged for consumers.”	1	0.19
“We agree the same”	1	0.19
“If he won’t eat something.”	1	0.19

## Data Availability

Data are contained within the article.
